# Miniaturized thermal acoustic gas sensor based on a CMOS microhotplate and MEMS microphone

**DOI:** 10.1038/s41598-022-05613-0

**Published:** 2022-02-01

**Authors:** Richard Hopper, Daniel Popa, Florin Udrea, Syed Zeeshan Ali, Phillip Stanley-Marbell

**Affiliations:** 1 Department of Engineering, University of Cambridge, Cambridge, CB3 0FA UK; 2 Flusso Limited, Cambridge, CB4 0DL UK

**Keywords:** Engineering, Nanoscience and technology, Physics

## Abstract

We present a miniaturised thermal acoustic gas sensor, fabricated using a CMOS microhotplate and MEMS microphone. The sensing mechanism is based on the detection of changes in the thermal acoustic conversion efficiency which is dependent on the physical properties of the gas. An active sensing element, consisting of a MEMS microphone, is used to detect the target gas while a reference element is used for acoustic noise compensation. Compared to current photoacoustic gas sensors, our sensor requires neither the use of gas-encapsulated microphones, nor that of optical filters. In addition, it has all the benefits of CMOS technology, including production scalability, low cost and miniaturization. Here we demonstrate its application for CO$_2$ gas detection. The sensor could be used for gas leak detection, for example, in an industrial plant.

## Introduction

Gas sensors play an increasingly important role in environmental monitoring due to elevated levels of atmospheric pollutants in urban areas, or the need for the safety monitoring of numerous industrial processes^1,2^. These requirements have fueled the demand for robust, low-cost, gas sensors that can detect hazardous levels of pollutants, including nitrogen dioxide (NO$_2$), carbon dioxide (CO$_2$) and volatile hydrocarbons such as benzene (C$_6$H$_6$)^1,2^. Current gas sensing technologies utilise a range of transduction effects, including gas-induced changes in the electrical, optical, physical and thermal properties of materials. Each technology has performance advantages/disadvantages specific to the detection of individual analytes, with no one universal approach^1,2^. A widely used sensing technology is the chemiresistor which utilises changes in the electrical conductivity of a semiconducting metal oxide (MOx) layer when it is exposed to an oxidising or reducing gas. These types of sensors are commonly used in low-cost, low-power applications, including portable air conditioning units^3^. Chemiresistors offer high sensitivity to organic compounds, however, they have several disadvantages including poor selectivity, environmental drift/poisoning and are insensitive to CO$_2$^4^. Optical sensors (including photoacoustic) can address some of these limitations, and are the tool of choice for monitoring CO$_2$, as well as a range of other gases^5^. Their operating mechanism is typically based on the detection of absorption lines in the mid-infrared (MIR) spectrum. They have enhanced selectivity compared to other approaches^6^ but are currently challenging to implement in a cost-efficient manner at chip-scale^5^. More recently, inexpensive alternatives have been reported based on CMOS thermal conductivity sensors which exploit changes in the thermal conductivity of the gas^7,8^.

Thermal acoustic systems are used to generate acoustic waves in a gas^9^. The effect was first investigated by F. Braun^10^ in 1898, who discovered that the electro-thermal modulation of a conductor could produce an acoustic wave in the surrounding air. An early example of such a device is the thermophone, developed in 1917 by H. D. Arnold and B. Crandall^11^, whose work helped establish the first theoretical understanding of its operation. Thermal acoustic systems are typically modelled as an electrically conducting plate, electro-thermally modulated by an alternating (AC) current^12–14^. These periodic electrical changes were thought to cause periodic fluctuations in the plate’s thermal energy and modulate the temperature (*T* [K]) of an air pocket adjacent to the plate in proportion to the periodical flow of heat ($\frac{Q}{\Delta t}$ [$\mathrm {\frac{J}{s}}$]). The contraction and expansion of air molecules in the heated zone was primarily attributed to the generation of an acoustic wave^12–14^. More recently, Daschewski^15^ has suggested that the thermal acoustic effect is caused by the exchange of momentum between the thermally modulated plate and gas molecules (rather than thermal expansion), with the molecules being rapidly ejected away from the thermally excited surface. To maximize the thermal-acoustic conversion efficiency (i.e. the ratio of acoustic to thermal power), it is important to minimize the thermal capacity (*C* [$\mathrm {\frac{J}{K}}$]) of the heat source^12–15^. Early approaches used freestanding metallic films and more recent approaches have used other types of structure, including ultra thin metallic films supported with porous silicon^16^ or CNT layers^17^. However, some of these approaches present manufacturability challenges and are not scalable.

In practical applications, thermal acoustic systems have previously been used as transducers and as components to convert thermal acoustic power into electrical energy^18^, with their thermal-acoustic conversion efficiency known to be influenced by the thermophysical properties of the gas^12–15^. However, little work has been done to study their application as actual gas sensors, including, e.g., for CO$_2$ gas sensing. CO_2_ is an asphyxiant gas, widely used in industrial processes and exposure to CO_2_ concentrations greater than 7% (70,000 to 100,000 ppm) may cause suffocation and unconsciousness within a few minutes to an hour. In this paper, we describe a miniaturised thermal acoustic sensor which utilizes a CMOS-based microhotplate chip and MEMS microphone and demonstrate its application for CO$_2$ gas sensing. The sensor benefits from the use of CMOS technology which offers excellent manufacturing scalability, low-cost and low-power consumption^19^.

## Results

**Figure 1:**
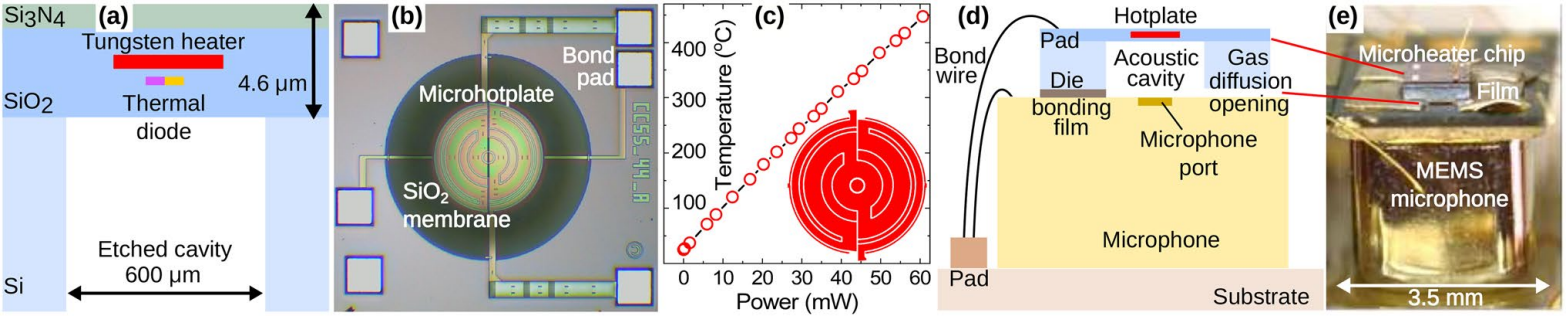
Device fabrication. (**a**) Structure of the CMOS microheater chip (not to scale) which employs a tungsten (W) heating element embedded in a $\sim$ 4.6 $\upmu$m thick silicon dioxide (SiO$_2$) membrane formed by deep reactive ion etching. A thermal diode is embedded in the centre of the heated membrane for temperature monitoring. (**b**) Optical image of the CMOS microheater chip, showing the multi-ring W heating elements and metal heatspreading plates embedded within a SiO$_2$ membrane. Chip size = 1.08 mm $\times$ 1.08 mm. (**c**) Temperature-power characteristics of the microheater up to 450 $^{\circ }$C at 60 mW. Inset: hotplate structure. (**d**) Schematic diagram showing the sensor’s construction. The microheater chip is mounted above the port of a MEMS microphone (TDK model ICS-40300) with a thermal acoustic cavity formed by the etched Si substrate. (**e**) Optical image of the fabricated thermal acoustic sensor, showing the microheater chip mounted on the MEMS microphone using die bonding film. The opening between the bonding films, to allow gas to diffuse into the thermal acoustic cavity, is visible (dark area).

Our miniaturised thermal acoustic gas sensor uses an in-house designed CMOS-based micro-hotplate chip, fabricated at a commercial MEMS foundry. A cross-section of the chip (not to scale) is shown in Fig. 1a, consisting of a circular 4.6 $\upmu$m thick SiO$_2$ membrane (600 $\upmu$m diameter), with an embedded tungsten (W) microheater (300 $\upmu$m diameter). Tungsten metallisation is chosen due to its very high melting point (3400 $^{\circ }$C) and low susceptibility to electromigration, thus ensuring stable electro-thermal performance^19,20^. The heater track has an electrical resistance of 39 $\Omega$ at room temperature. The membrane is formed by deep reactive ion etching (DRIE) and thermally isolates the heater from the Si substrate. The microheater chip also incorporates an p$^+$–n$^+$ junction thermal diode, embedded in the SiO$_2$ membrane, which is used to monitor the operating temperature. The forward bias voltage of the diode has a linear response to temperature to over 600 $^{\circ }$C^21^, with a temperature coefficient of − 1.34 mV/$^{\circ }$C. Figure 1b shows an optical image of the fabricated chip, showing the meandering heater track design (chip dimensions: 1.08 mm $\times$ 1.08 mm $\times$ 0.38 mm). The temperature-power characteristics of the heater are shown in Fig. 1c, determined by the thermal diode. The heater reaches a temperature of 300 $^{\circ }$C at an operating power of only 38 mW, and is characterised by a fast ($\sim$ 9 ms) thermal time constant^20^.

To construct our sensor, we mounted the microheater chip above the port of an analogue MEMS microphone (TDK model ICS-40300), in a simple, miniature (non-resonant) configuration^5^, as depicted in Fig. 1d. The microphone has an extended frequency response of 6 Hz–20 kHz and a signal-to-noise ratio (SNR) of 63 dBA. Die mounting pads were used to mechanically secure the microheater chip to the microphone’s substrate. An acoustic cavity is thereby formed between the etched membrane of the microheater chip and the microphone port. To allow gas to diffuse into the cavity, a small opening (dimensions: $\sim$ 500 $\times$ 50 $\upmu$m) was created between the die mounting pads. For characterisation, both the microheater and microphone were mounted on a TO-8 metal package. An optical image of the fabricated sensor is shown in Fig. 1e.

**Figure 2:**
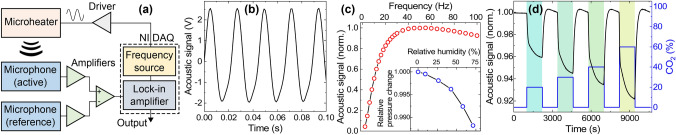
Sensor interfacing. (**a**) Block diagram of the experimental setup. The microheater is electrically modulated using a custom current drive circuit. Signals from the active and reference MEMS microphones are amplified and their differential signal digitised using a National Instruments data acquisition unit, with a software based lock-in amplifier used to help remove noise. (**b**) Modulated acoustic signal showing the pressure changes which occur during electro-thermal modulation of the sensor at a frequency of 43 Hz. (**c**) Acoustic frequency response of the sensor, showing the rise in the acoustic signal with modulation frequency and gradual tail off in the response due to thermal inertia of the membrane which limits the thermal modulation depth. Inset: relative change in pressure with humidity. (**d**) Response of the sensor to pulses of ~ 20–60 % CO$_2$ gas concentrations showing the drop in the thermal acoustic signal with increasing CO$_2$ concentration.

To interface the sensor, we used a custom heater current drive and amplification electronics, as shown in Fig. 2a. The electronics were interfaced to a National Instruments (NI) data acquisition (DAQ) card, so that modulation and data acquisition could be done automatically using LabVIEW software. The microheater was sinusoidally modulated using a current source with a peak current of 30 mA at a frequency of 43 Hz, corresponding to a peak temperature of $\sim$ 320 $^{\circ }$C. To compensate for background acoustic noise, the signal from a second ’reference’ microphone (acoustically isolated from the microheater), was subtracted from the active thermal acoustic signal, after voltage amplification (100$\times$). The resulting differential modulated acoustic signal, generated by the thermal acoustic effect, is shown in Fig. 2b. A software based lock-in amplifier, with a time constant of 1 s, was used to process the recovered waveform and extract the amplitude of the acoustic response. The acoustic response across a range of modulation frequencies is plotted in Fig. 2c. As the heater modulation frequency (*f* [Hz]) increases [from near direct current (DC)], the level of the acoustic signal rises, as the rate of heat flow into the gas increases, peaking at around 43 Hz. At higher modulation frequencies, the microheater’s temperature modulation depth ($\Delta T$ [K]) decreases due to its thermal inertia (or thermal effusivity, a measure of the rate at which it absorbs or releases thermal energy^22^), as shown in Fig. 2c, causing a shallow drop-off in the strength of the acoustic signal. The thermal acoustic sensor shows a change of around 1% in measured pressure from 0 to 70% relative humidity, as showed in the inset of Fig. 2c.

For gas tests, we mounted the sensor in a stainless steel chamber kept at 25 $\pm 1\;^{\circ }$C ambient temperature by a Dri-bloc™ heater. Mass flow controllers (MFCs) were used to regulate the flow of gas into the chamber. Gas tests were done with CO$_2$ diluted in zero grade air at 0% relative humidity. The total flow of gas through the system was kept constant at 200 mL/min. The response of the sensor exposed to CO$_2$ concentrations varying from 0 to 60% is shown in Fig. 2d. The sensor gives a relative change in amplitude response of 0.13% per 1% change in CO$_2$ concentration, giving a limit of detection (3$\sigma$) of 0.14% CO$_2$. These values are comparable to current state-of-the-art thermal conductivity based sensors^7,8^, and could be further improved by minimizing the thermal heat capacity of the heat source to reduce the heating time, i.e., maximise the transfer of kinetic energy into the gas, ultimately boosting the thermal acoustic signal. There is a small recovery period after each gas exposure, thought to be due to thermal stabilisation of the sensor.

**Figure 3:**
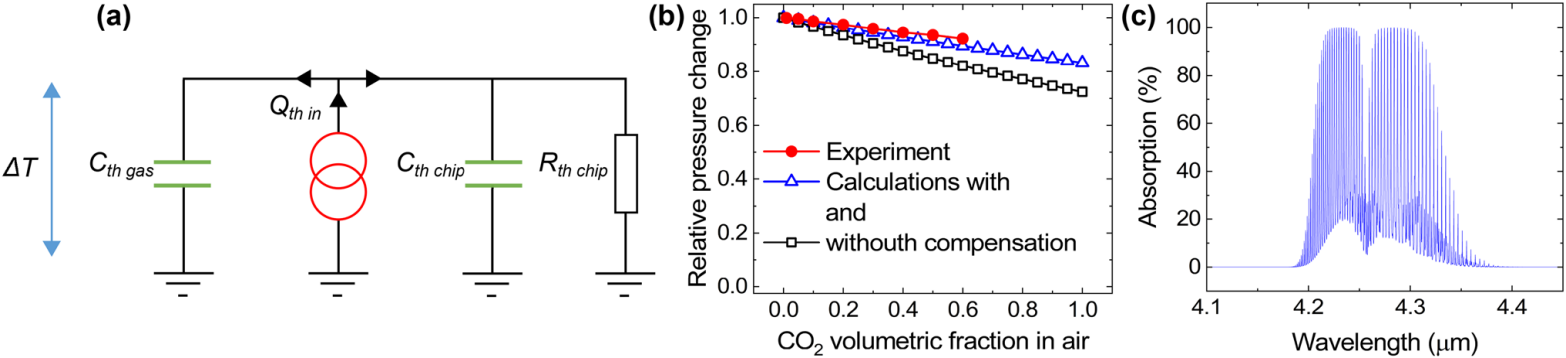
Numerical analysis. (**a**) Simplified thermal model illustrating the heat flow from the microheater into the gas and chip due to their thermal capacities $C_{th}$ and resistance $R_{th}$ of the chip under DC heating. A temperature rise Δ*T* occurs on the microheater due to the flow of heat. (**b**) Experimental vs. numerical calculations showing the relative pressure change in the thermal acoustic cavity with varying volumetric fractions of CO$_2$, modelled with and without compensation for the photoacoustic effect. (**c**) Mid-infrared absorption spectrum with 20% CO$_2$ in air at 0.1 mm absorption length, under standard conditions (296 K, 1 atm).

In order to better understand the response dynamics of our sensor, we performed numerical calculations based on a standard thermal circuit model^23^, accompanied by thermodynamics^15,22^. Thermal systems can be conveniently represented as electrically equivalent thermal circuits. Figure 3a shows a simplified electrical equivalent thermal circuit for our thermal acoustic sensor where the gas’s and chip’s thermal capacitances $C_{th} = Q/ \Delta T$ [$\mathrm {\frac{J}{K}}$] represent a measure of the heat needed to produce a change in temperature. The microheater chip’s thermal resistance *R*_th chip_ is also shown, signifying the temperature change per unit Watt of electrical power under DC heating. A current source *I* [A] is used to represent the heat flow $Q/ \Delta t$ [W] that generates a temperature rise $\Delta T$ [K] across the gas, equivalent to a voltage. In our case, the two gas volumes, either side of the heater, have the same thermal properties, and thus the same $C_{th}$. The heater is modulated by electrical excitation [$I(t)= I_0 \cdot sin(\omega \cdot t)$, with $I_0$ the current magnitude, and $\omega =2\cdot \pi \cdot f$ the angular frequency, where *f* [Hz] is the thermal modulation frequency], closely related to the supplied electrical power ($I\cdot V$ [W] proportional to $Q/\Delta t$), causing a temperature increase. Gas molecules receive thermal energy as momentum resulting in them being directed away from the heated surface^15^, with the velocity at which their ejection occurs dependent on the heating rate and therefore frequency of thermal modulation^15,22,23^.

A further mathematical description of our thermal acoustic system can be derived by thermodynamics^15,22,23^. The average change in acoustic pressure $\Delta p$ [Pa] generated in our thermally modulated system of constrained volume *V* [m$^3$] is proportional to the average change of internal thermal energy of the gas $\Delta U$ [J], given by

1 $$\begin{aligned} \Delta p( f ) = \frac{ \Delta U ( f ) }{ V } \cdot z ~\mathrm {[Pa]}, \end{aligned}$$

where *z* is a constant to account for acoustic losses through the small opening of the cavity which increases its effective volume. From Fig. 3a, the heat $\Delta Q$ [J] supplied into the gas, during a heating period of time $t_{th}(f)$ [s], can be written as

2 $$\begin{aligned} \Delta Q(f) = 2 \cdot \pi \cdot f \cdot \Delta T \cdot C_{th} (f) \cdot t_{th} (f)~\mathrm {[J]}, \end{aligned}$$

where $\Delta T$ [K] is the average change in temperature during the heating period $t_{th}$ [s], and $C_{th}$ [$\mathrm {\frac{J}{K}}$] is the thermal capacity of the gas, given by

3 $$\begin{aligned} C_{th} (f) = d_{th} (f) \cdot A \cdot \rho \cdot c~\mathrm {[\mathrm {\frac{J}{K}}]}, \end{aligned}$$

where the thermal penetration depth $d_{th}(f) = \sqrt{ \frac{ \alpha _{diff} }{ 2 \cdot \pi \cdot f } }$ [m] is a function of *f* and the thermal diffusivity $\alpha _{diff} = k \cdot \rho / c$ [$\mathrm {\frac{m^2}{s}}$] of the gas, *A* [m$^2$] is the area of the heat source, and *k* [$\mathrm {\frac{W}{m \cdot K}}$], $\rho$ [$\mathrm {\frac{kg}{m^3}}$] and *c* [$\mathrm {\frac{J}{kg \cdot K}}$] are the thermal conductivity, density and specific heat capacity of the gas, respectively. Substituting for $d_{th}$ and rearranging we get

4 $$\begin{aligned} C_{th}(f) = \frac{ e \cdot A }{ \sqrt{ 2 \cdot \pi \cdot f } }~\mathrm {[\mathrm {\frac{J}{K}}]}, \end{aligned}$$

where $e = \sqrt{ k \cdot \rho \cdot c }$ [$\mathrm {\frac{J}{m^2 \cdot K \cdot \sqrt{s}}}$] is the thermal effusivity of the gas. The acoustic pressure change can now be expressed as

5 $$\begin{aligned} \Delta p( f ) = \frac{ \sqrt{ 2 \cdot \pi \cdot f } \cdot \Delta T \cdot e \cdot A \cdot t_{th} ( f) \cdot z}{ V }~\mathrm {[Pa]}. \end{aligned}$$

Momentum is propagated by the collision of the gas particles, as they move away from the heated surface, increasing the pressure. As the heater’s temperature decreases, the velocity of the gas particles decreases, causing them to return to their initial thermodynamic state^15^. At high thermal modulation frequencies *f*, the gas does not expand in volume due to self-heating and does not store the supplied $\Delta Q(f)$. In this scenario, a particle-velocity wave in the adjacent gas is formed which propagates at the speed of sound. If the heating time is long (seconds), the mechanism of operation is different and the gas layer close to the heated surface has time to store the kinetic energy as heat, increasing in volume. $\Delta Q(f)$ supplied to the gas is then dissipated by convective gas flow, thermal diffusion and radiation^15,22,23^.

We use the model described above to calculate the relative change in pressure, caused by a gradual increase (from 0 to 100%) of CO$_2$ concentration in air, with respect to air. Figure 3b (black line) shows a maximum $\sim$ 28% relative decrease in pressure for 100% CO$_2$ with respect to that of air. When compared to the experimental data, plotted in Fig. 3b (red line), a deviation ($\sim$ 0.16 slope) from the numerical calculations can be observed. In this experiment, our microheater has a dual-effect. It provides the thermal modulation profile needed for thermal acoustic conversion, and, at the same time, acts as a MIR thermal source, responsible for an additional photoacoustic effect in the cavity^24^. With increasing the CO$_2$ concentration, the thermal acoustic effect, described above, is responsible for a decrease in the acoustic pressure, which is related to the CO$_2$ effusivity with respect to that of air. On the other hand, a competing photoacoustic effect is responsible for an increase in the acoustic pressure, proportional to the CO$_2$ absorption cross section ($\sigma (\lambda )$ [cm$^2$], with $\lambda$ [m] the optical wavelength) in the MIR (significantly higher than that of air)^24^. An expression for the photoacoustic signal, which is proportional to the optical power, can be derived from the Beer–Lambert law^24^:

6 $$\begin{aligned} P \approx P_0 \cdot \sigma (\lambda ) \cdot N \cdot l ~\mathrm {[W]}, \end{aligned}$$

where $P_0$ and *P* are the optical power before and after the photoacoustic cell, respectively, *N* [cm$^{-3}$] is the number of absorbing molecules per cubic centimeter, and *l* [cm] the absorption path length. In order to account for the additional photoacoustic effect, we use HITRAN data^25^ to calculate the optical absorption A ($\lambda$) in the sensor’s cavity. An absorption spectrum for 20% CO$_2$ in air is shown in Fig. 3c for a 1 mm absorption length. For our microhotplate we assume a grey body emitter profile G ($\lambda$) with a defined 300 $^{\circ }$C temperature^20^. The absorbed power is calculated by numerical integration of the absorption data with that of the microheater’s profile:

7 $$\begin{aligned} P_{abs} = \int A(\lambda ) \cdot G(\lambda ) d\lambda ~\mathrm {[W]}, \end{aligned}$$

which is then used to adjust $\Delta Q$ [J] supplied into the gas to account for the additional photoacoustic effect. The new relative change in pressure is plotted in Fig. 3b (blue line) showing a reduced ($\sim$ 0.04 slope) deviation from the experimental data. It is important to note that most current photoacoustic gas sensors employing MEMS heaters as MIR sources use MEMS microphones encapsulated with the target gas, thus minimizing any direct transfer of thermal to kinetic energy from the heater^5,26^. In our sensor, the heater is in direct contact with the gas to maximise the direct energy transfer.

## Discussion

In this paper, we have reported on a novel technique for gas sensing, exploiting changes in the thermal acoustic conversion efficiency of gases. By combining a CMOS MEMS microhotplate, as a thermal source, and a MEMS microphone, we demonstrate an ultra-compact gas sensor with a limit of detection of 0.14% to CO$_2$. We provide a theoretical model, to accompany the experimental results, showing the sensor's response is mostly dependent on the thermal effusivity of the gas, thus allowing for simple sensor design and optimization. We believe the thermal acoustic technique is a simple and cost-effective approach that can be used for a variety of applications. A further refinement would be to investigate approaches to decouple the thermal and photo acoustic effects respectively, for example, by minimising IR emission.

## Methods

The micro-hotplate was fabricated using a commercial 1 $\upmu$m silicon-on-insulator (SOI)-CMOS process on 6 inch 375 $\upmu$m thick silicon (Si) wafers. Deep Reactive Ion Etching (DRIE) was used to form the membrane; utilising the first silicon dioxide layer as an etch-stop. The DRIE etching process creates near vertical side-walls. Si$_3$N$_4$ is used as a passivation layer. The microheater area is ~ 0.07 mm^2^.

The microphone (model ICS-40300, acting as the acoustic sensor) was packaged in a surface-mount package, with a metal cap on top of a substrate layer (dimensions: 4.72 mm $\times$ 3.76 mm $\times$ 3.5 mm). The microheater was driven using a sinusoidal voltage signal via a buffer amplifier while op-amp based amplifiers (10 $\times$ voltage gain) were used to amplify the microphonic signals prior to digitisation. The thermodiode was constant current biased at 100 $\upmu$A. The sensor was interfaced to a National Instruments DAQ card (model NI USB-6353), so that control and data acquisition could be done automatically using LabVIEW software. A lock-in amplifier was implemented in software to process the recovered waveform. The effect of background acoustic noise was reduced by digital noise cancellation and integration.

Data from additional sensors was collected during the gas tests, including pressure, temperature, humidity data from a Bosch BME680 sensor and CO_2_ concentration data from a SCD30 optical sensor. The sensors were interfaced for data logging using a Warp RevC board^27^.
